# Bone metastases in newly diagnosed patients with thyroid cancer: A large population-based cohort study

**DOI:** 10.3389/fonc.2022.955629

**Published:** 2022-08-12

**Authors:** Ruiguo Zhang, Wenxin Zhang, Cailan Wu, Qiang Jia, Jinyan Chai, Zhaowei Meng, Wei Zheng, Jian Tan

**Affiliations:** ^1^ Department of Nuclear Medicine, Tianjin Medical University General Hospital, Tianjin, China; ^2^ Department of Nuclear Medicine, Tianjin Fourth Central Hospital, Tianjin, China

**Keywords:** thyroid cancer, bone metastases, epidemiology, population-based study, histologic subtype, SEER (Surveillance Epidemiology and End Results) database

## Abstract

**Background:**

Population-based estimates of the incidence and prognosis of bone metastases (BM) stratified by histologic subtype at diagnosis of thyroid cancer are limited.

**Methods:**

Using multivariable logistic and Cox regression analyses, we identified risk factors for BM and investigated the prognostic survival of BM patients between 2010 and 2015 *via* the Surveillance, Epidemiology, and End Results (SEER) database.

**Results:**

Among 64,083 eligible patients, a total of 347 patients with BM at the time of diagnosis were identified, representing 0.5% of the entire cohort but 32.4% of the subset with metastases. BM incidence was highest (11.6%) in anaplastic thyroid cancer (ATC), which, nevertheless, was highest (61.5%) in follicular thyroid cancer (FTC) among the subset with metastases. The median overall survival among BM patients was 40.0 months, and 1-, 3-, and 5-year survival rates were 65.2%, 51.3%, and 38.7%, respectively. Compared with papillary thyroid cancer (PTC), FTC (aOR, 6.33; 95% CI, 4.72–8.48), medullary thyroid cancer (MTC) (aOR, 6.04, 95% CI, 4.09–8.92), and ATC (aOR, 6.21; 95% CI, 4.20–9.18) significantly increased the risk of developing BM. However, only ATC (aHR, 6.07; 95% CI, 3.83–9.60) was independently associated with worse survival in multivariable analysis. Additionally, patients with BM alone (56.5%) displayed the longest median survival (66.0 months), compared with those complicated with one extraskeletal metastatic site (lung, brain, or liver) (35.2%; 14.0 months) and two or three sites (8.3%; 6.0 months). The former 5-year overall survival rate was 52.6%, which, however, drastically declined to 23.0% in patients with one extraskeletal metastatic site and 9.1% with two or three sites.

**Conclusion:**

Closer bone surveillance should be required for patients with FTC, MTC, and ATC, and extraskeletal metastases at initial diagnosis frequently predict a poorer prognosis.

## Introduction

Preemptive surveillance for the presence of lymph node and lung metastases is the standard-of-care in thyroid cancer patients (1, 2). Although thyroid cancer is predisposed to bone metastases (BM), the existent data are limited to older, retrospective, single-institution studies with small cohorts (3–5) or large cohorts but mainly focused on differentiated thyroid cancer (DTC) (6). Additionally, the occurrence of BM is less well studied, which, in the literature varies widely (7, 8), occurring in 2%–15% of pooled DTC series and 1.4%–7% in papillary thyroid cancer (PTC) patients. Also, large population-based estimates relating to the incidence of BM at diagnosis of thyroid cancer based on histologic subtype or a number of extraskeletal metastases are limited.

In a study of 245 DTC patients with BM, 78% presented with or developed ≥1 skeletal-related event after initial detection of BM, and the median time from identification of BM to first skeletal-related events was less than 5 months (9). A recent study conducted by Vuong et al. (10) revealed that follicular thyroid carcinoma (FTC) and medullary thyroid carcinoma (MTC) at diagnosis were predisposed to BM compared with PTC and anaplastic thyroid cancer (ATC) *via* the Surveillance, Epidemiology, and End Results (SEER) database. Although the entire prognosis of thyroid cancer is good in comparison to other cancers, leading to a survival rate of 93% at 10 years (1, 2), the survival rate in patients with BM obviously declines, with 5- and 10-year survival rates of 61% and 27% from the initial BM diagnosis in DTC patients (11), and 10-year 15% in pooled DTC patients (4). On the other side, interestingly, metastases to bone alone frequently show an improved outcome compared to liver, lung, and brain alone (10). However, BM may also coexist with other distant metastases. The outcomes drastically decline in patients exhibiting multiorgan metastasis, with a 5-year survival rate of 15.3%, compared to 77.6% of patients with single-organ metastasis (12). However, regarding the BM among patients with newly diagnosed thyroid cancer, the prognosis based on their histologic subtype, especially for MTC/ATC and the number of extraskeletal metastases, is unclear.

Early identification of factors predicting BM and survival prognosis at diagnosis of thyroid cancer is of great value. Various retrospective studies tried to identify factors independently predicting the prognosis of DTC patients with BM (13–15). Nevertheless, many of them lead to inconclusive results (8), since cohorts were often assembled over multiple decades during which both histopathologic evaluation and clinical management have evolved. More recently, Qi et al. have identified predictors of developing BM in patients with DTC using population-based data, but regretfully, the rare ATC and MTC subtypes were not included (6).

This study was to use the SEER database to characterize the incidence proportion of BM at the diagnosis of thyroid cancer on a population-based level. We also sought to quantify survival estimates and to explore clinical and sociodemographic predictors of poorer survival among patients with BM in newly diagnosed thyroid cancer.

## Patients and methods

### Study population

**Figure 1:**
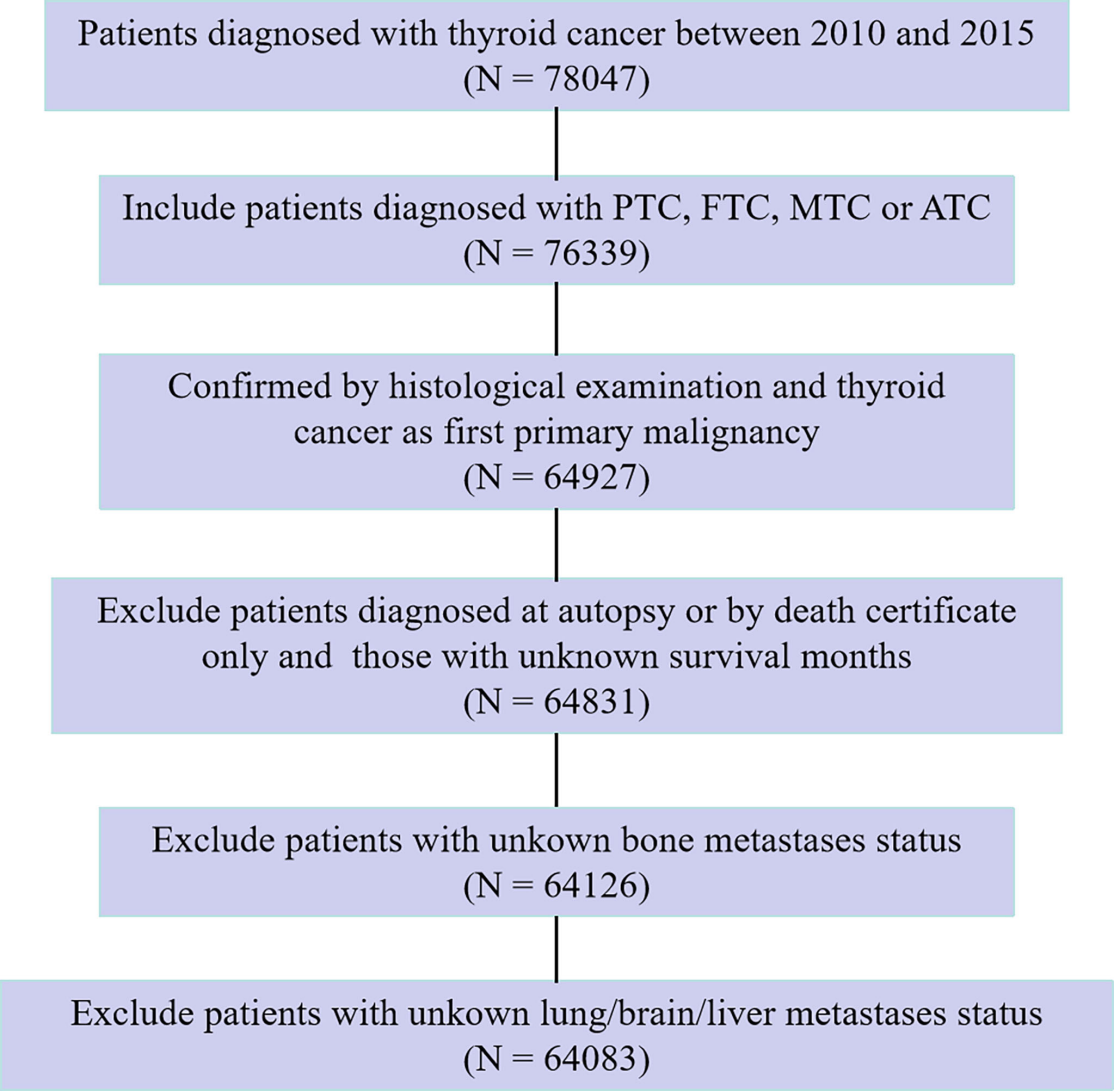
Flow chart for patient selection procedure of the SEER dataset.

In the SEER database, data from patients histologically diagnosed with thyroid cancer and as a first primary malignancy between 2010 and 2015 were extracted for this study. In the present study, tumor histology was limited to four categories according to the International Classification of Disease for Oncology, 3rd Edition (ICD-O-3) histological codes: PTC (8050, 8260, 8340–8344, 8450–8460), FTC (8290, 8330–8335), MTC (8345, 8510–8513), and ATC (8020–8035). Hence, thyroid cancer in this study specifically refers to the four abovementioned histologic subtypes. We excluded patients who were diagnosed at autopsy or *via* death certificate only, as well as those who had an unknown follow-up period eligible for survival analysis. In addition, patients for whom the presence or absence of brain/bone/liver/lung metastasis at diagnosis was unknown were also excluded. After exclusion criteria were fulfilled, the study cohort finally comprised 64,083 eligible and statistically analyzable patients. The flow chart of the patient selection procedure in this study is shown in **Figure 1**.

Demographic variables in the analysis included patient age at diagnosis, sex, race, and marital status. Clinical and pathologic characteristics included tumor size, lymph node involvement status, surgical and radiotherapy procedures, histologic subtypes, and the number of extraskeletal metastases to any distant site (lung, brain, and liver). In this study, according to the SEER database, the race was categorized as black, white, other, or unknown. Radiotherapy procedures were divided into no, yes (radiation and/or isotopes), refused, or unknown. Tumor size was categorized as follows: ≤10, 11 to 19, 20 to 39, ≥40 mm, or unknown. Age at diagnosis was grouped as <20, 20 to 39, 40 to 59, or ≥60 years. Lymph node involvement status was recorded as no lymph node metastases (no), lymph node metastases (yes), or *N_X_* (unknown). With regard to surgical procedure, RX Summ–Surg Prim Site (1998+) code of 50 was categorized as total thyroidectomy, 10–40 as others (including local tumor destruction (code 13), removal of less than a lobe (codes 25–27), lobectomy and/or isthmectomy (codes 20–23), removal of a lobe and partial removal of the contralateral lobe (code 30), and subtotal or near-total thyroidectomy (code 40)), 00 as no surgery (no) and 80, 90, and 99 as unknown. As for marital status, in the SEER database, we classified single and unmarried or domestic partners as “unmarried” and married, divorced, widowed, and separated as “married.”

Overall survival (OS) was defined as the time between diagnosis and death from any cause, and thyroid cancer-specific survival (TCSS) was defined as the time between diagnosis and death from thyroid cancer. Additionally, patients based on the number of extraskeletal metastatic sites (lung, brain, and liver), as well as histologic subtype, were separately analyzed. All patients included were staged using the seventh edition of the AJCC TNM staging system.

### Statistical analyses

The Pearson’s *χ* ^2^ test was used to compare variables between the entire cohort of patients with BM and those without. The variables in the model included age at diagnosis, sex, race, marital status, tumor size, lymph node involvement status, and histologic subtypes. Variables with *p-*values less than.05 in the univariate analysis were further integrated into the multivariate logistic regression analysis. The predictors associated with the occurrence of BM at diagnosis of thyroid cancer were then identified.

Survival estimates were obtained using the Kaplan–Meier method with survival curves compared using the log-rank test among patients with metastases at diagnosis of thyroid cancer. Besides the variables as in the logistic regression model described herein, clinical factors affecting the patients’ survival, such as surgical and radiotherapy procedures, and the number of extraskeletal metastases, were also included in the survival analysis. For the variables with *p-*values less than.05 in the log-rank test, we further performed multivariable Cox proportional hazard regression to analyze the association between potential predictors and all-cause mortality and thyroid cancer-specific mortality. All data were analyzed using SPSS (version 26, IBM Corporation, Armonk, New York, USA) with a two-sided test, and *p*-values less than.05 were considered to be statistically significant.

## Results

### Incidence

The number and incidence proportions of patients with thyroid cancer and bone/lung/brain/liver metastases identified at diagnosis are provided in **Table 1**, as stratified by tumor subtype. Among the entire cohort, 347 patients presented with BM, representing 0.5% of the entire study population. Among the entire cohort by subtype, ATC had the highest incidence proportion of BM (11.6%), followed by MTC. While among the subset with metastases, FTC had the highest proportion (61.5%).

**Table 1:** Incidence proportion of patients with bone metastases identified at diagnosis of thyroid cancer by subtype.

		Patients, No., and incidence proportion (%) of organ-specific metastases among the entire cohort by subtype					Patients and No. based on extraskeletal metastatic sites to the lung, brain, and liver among patients with metastases			Patients, No., and incidence proportion (%) of bone metastases among the subset with metastases.
Subtype	With thyroid cancer	With metastases	With bone metastases	With lung metastases	With liver metastases	With brain metastases	0	1	2 or 3	
PTC	58,385	588 (1.0)	147 (0.3)	348 (0.6)	19 (0.0)	18 (0.0)	233	346	19	147 (25)
FTC	4,252	187 (4.4)	115 (2.7)	90 (2.1)	10 (0.2)	9 (0.2)	88	89	10	115 (61.5)
MTC	1,033	95 (9.2)	37 (3.6)	32 (3.1)	43 (4.2)	7 (0.7)	28	54	13	37 (38.9)
ATC	413	202 (48.9)	48 (11.6)	153 (37.0)	16 (3.9)	18 (4.4)	36	146	20	48 (23.8)
All subtypes	64,083	1,072 (1.7)	347 (0.5)	623 (1.0)	88 (0.1)	52 (0.1)	375	635	62	347 (32.4)

PTC, papillary thyroid cancer; FTC, follicular thyroid cancer; MTC, medullary thyroid cancer; ATC, anaplastic thyroid cancer.

For each histologic subtype, the most common metastatic sites at the time of diagnosis were the lung in PTC and ATC, bone in FTC, and liver in MTC subtypes. In advance, patients with ATC at initial diagnosis had the highest metastatic proportion (48.9%), with metastases occurring in 202 of the total 413 patients.

### Clinical characteristics and risk factors for patients with bone metastases

Baseline characteristics and correlation of the entire cohort and patients with bone metastasis at diagnosis of thyroid cancer are demonstrated in **Table 2**. The median age of the patients with BM was 64 (interquartile range, 55–72) at the moment of diagnosis. In univariable analysis, older age, male gender, black race, married, large tumor size, lymph node metastases, and ATC/FTC/MTC histology were significant risk factors for the presence of BM at presentation.

**Table 2:** Baseline demographic and clinical characteristics and risk factors of bone metastases at presentation.

Variable	Overall cohort patients (*n* = 64,083)	With metastases (*n* = 1,072)	With bone metastases (*n* = 347) and incidence proportion among the entire cohort (%)	Pearson’s *χ* ^2^ value	*p-*value
**Age at diagnosis (year)**					
<20	1,350	32	0 (0)	286.58	**<0.001**
20–39	17,151	106	26 (0.2)		
40–59	29,539	295	100 (0.3)		
≥60	16,043	639	221 (1.4)		
Median (IQR)	49 (38–60)	64 (55–72)			
**Sex**					
Male	14,654	481	158 (1.1)	101.62	**<0.001**
Female	49,429	591	189 (0.4)		
**Race**					
Black	4,535	104	54 (1.2)	52.19	**<0.001**
White	51,305	808	238 (0.5)		
Other	7,325	155	55 (0.8)		
Unknown	918	5	0 (0)		
**Marital status**					
Married	46,117	807	274 (0.6)	10.67	**0.005**
Unmarried	14,018	226	63 (0.4)		
Unknown	3,948	39	10 (0.5)		
**Tumor size (mm)**					
≤10	24,684	89	22 (0.1)	906.24	**<0.001**
11–20	17,464	127	49 (0.3)		
20–40	14,221	229	69 (0.5)		
≥40	6,347	453	142 (2.2)		
Unknown	1,367	174	65 (4.8)		
**Lymph node metastases**					
No	48,224	365	172 (0.4)	242.51	**<0.001**
Yes	15,136	620	146 (1.0)		
Unknown	723	87	29 (4.0)		
**Histologic subtype**					
PTC	58,385	588	147 (0.3)	1,579.31	**<0.001**
FTC	4,252	187	115 (2.7)		
MTC	1,033	95	37 (3.6)		
ATC	413	202	48 (11.6)		

PTC, papillary thyroid cancer; FTC, follicular thyroid cancer; MTC, medullary thyroid cancer; ATC, anaplastic thyroid cancer; IQR, interquartile range.

**Figure 2:**
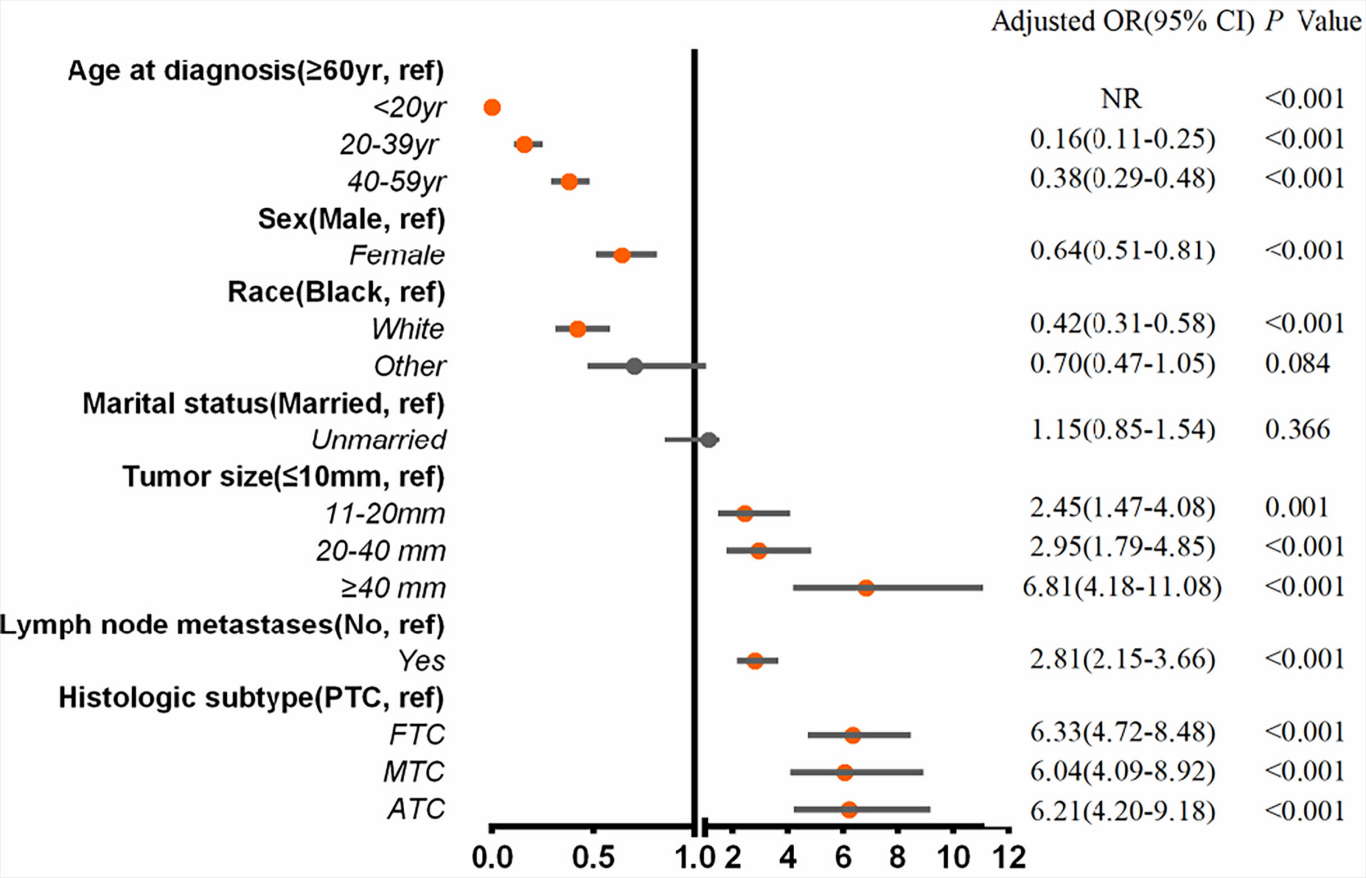
Multivariable predictors for the presence of bone metastases at diagnosis of thyroid cancer. aOR, adjusted odds ratio; CI, confidence interval; yr, years.

Multivariable logistic regression analysis was applied to assess the risk variables for the presence of BM among the entire cohort of patients (**Figure 2**). Tumor sizes of 11 to 19 mm (adjusted odds ratio (aOR), 2.45; 95% CI, 1.47–4.08; *p* = .001), tumor sizes of 20 to 39 mm (aOR, 2.95; 95% CI, 1.79–4.85; *p* <.001), tumor sizes of ≥40 mm (aOR, 6.81; 95% CI, 4.18–11.08; *p* <.001), lymph node metastases (aOR, 2.81; 95% CI, 2.15–3.66; *p* <.001), FTC (aOR, 6.33; 95% CI, 4.72-8.48; *p* <.001), MTC (aOR, 6.04; 95% CI, 4.09–8.92; *p* <.001), and ATC (aOR, 6.21; 95% CI, 4.20–9.18; *p* <.001) were associated with significantly greater odds of developing BM in newly diagnosed thyroid cancer. Meanwhile, age ≤20 years (aOR, 0; *p* <.001), age 20 to 39 years (aOR, 0.16; 95% CI, 0.11–0.25; *p* <.001), age 40 to 59 years (aOR, 0.38; 95% CI, 0.29–0.48; *p* <.001), female gender (aOR, 0.64; 95% CI, 0.51–0.81; *p* <.001), and white race (aOR, 0.42; 95% CI, 0.31–0.58; *p* <.001) were less frequently associated with having BM at diagnosis.

### Survival

**Figure 3:**
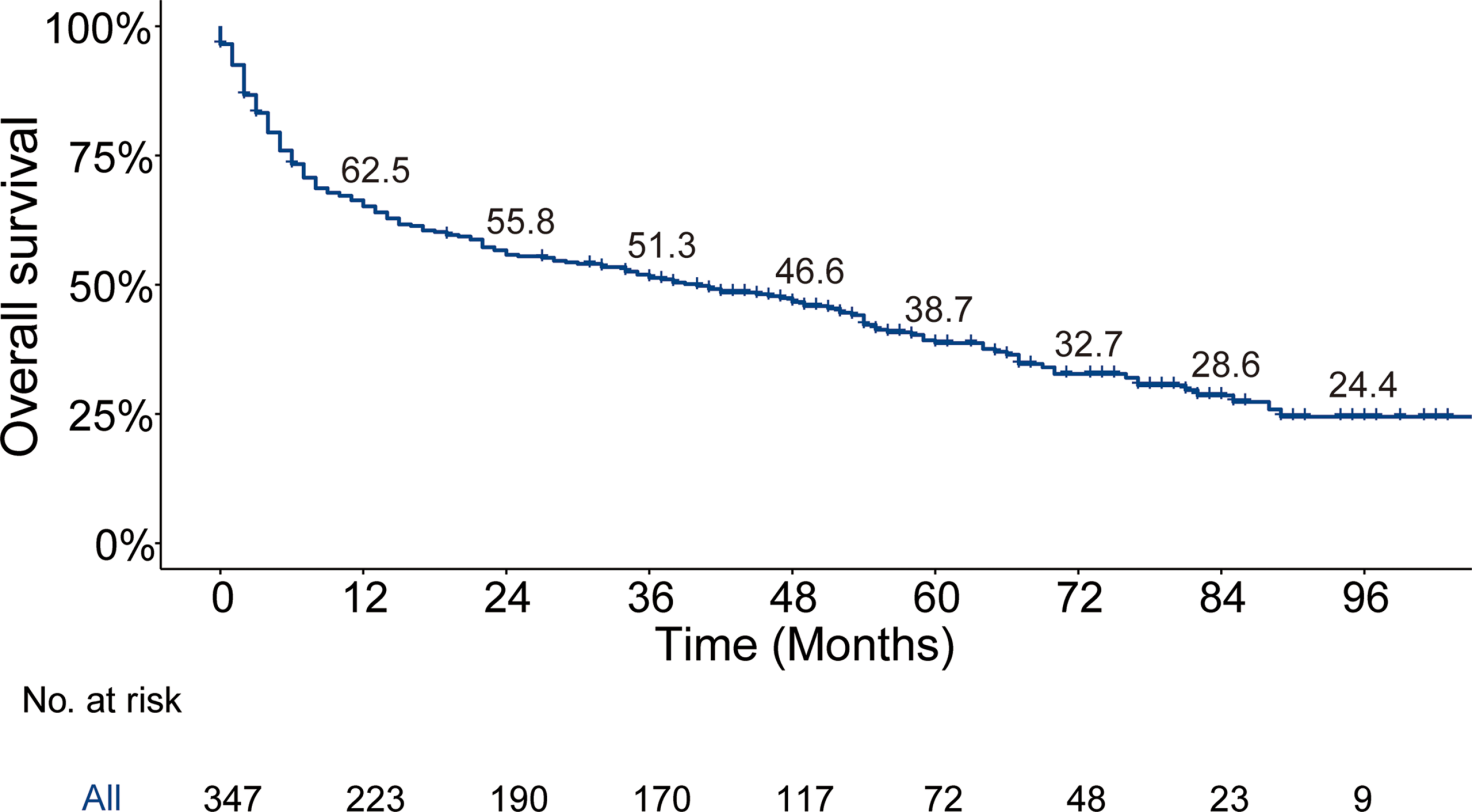
Kaplan–Meier’s overall survival curve and the corresponding annual survival rate in patients with bone metastases at diagnosis of thyroid cancer. Median overall survival, 40 months.

The median follow-up time for the entire cohort (*n* = 64,083) until censoring or death was 65 months (range, 0 to 107 months) and 35 months (range, 0 to 107 months) for patients with BM (*n* = 347). The median OS for patients with BM at diagnosis of thyroid cancer (*n* = 347) was 40.0 months (95% CI, 28.0–52.5), and 1-, 3-, and 5-year OS rates were 65.2%, 51.3%, and 38.7%, respectively, which is depicted by the Kaplan–Meier curve in **Figure 3**. The Kaplan–Meier survival analysis for all-cause mortality and thyroid cancer-specific mortality among patients with BM at diagnosis is listed in **Table 3**. The results of the univariable analysis demonstrated that age at diagnosis, tumor size, surgical procedure, lymph node metastases, radiotherapy, histologic subtype, and the number of extraskeletal metastatic sites were all associated with survival outcomes (*p* <.001). However, we did not find the impact of gender, race, and marital status on the OS and TCSS.

**Table 3:** Kaplan–Meier survival analysis for all-cause mortality and thyroid cancer-specific mortality among patients with bone metastases at diagnosis of thyroid cancer.

Variable	Entire cohort patients (*n* = 64,083)	With bone metastases (*n* = 347)	All-cause mortality			Thyroid cancer-specific mortality		
			Median OS of patients with bone metastases [month (95% CI)]	*χ* ^2^ value	*p*-value	Median TCSS of patients with bone metastases [month (95% CI)]	*χ* ^2^ value	*p*-value
**Age at diagnosis (year)**								
<20	1,350	0	NR	16.73	**<0.001**		12.07	**0.002**
20–39	17,151	26	64.0 (34.4–93.6)			85.0 (43.5–126.5)		
40–59	29,539	100	65.0 (45.3–84.7)			NR		
≥60	1,6043	221	23.0 (12.4–33.6)			36.0 (18.5–53.5)		
**Sex**								
Male	14,654	158	38.0 (20.9–55.1)	0.11	0.738		0.07	0.797
Female	49,429	189	41.0 (26.1–56.0)			54.0 (42.2–65.8)		
**Race**								
Black	4,535	54	51.0 (29.0–73.0)	3.75	0.153		3.90	0.143
White	51,305	238	28.0 (12.8–43.2)			45.0 (22.1–67.9)		
Other	7,325	55	55.0 (35.6–74.4)			NR		
Unknown	918	0	NR			NR		
**Marital status**								
Married	46,117	274	38.0 (23.9–52.1)	0.76	0.684		0.39	0.825
Unmarried	14,018	63	49.0 (12.3–85.7)			58.0 (34.8–81.2)		
Unknown	3,948	10	NR			NR		
**Tumor size (mm)**								
≤10	24,684	22	NR	53.00	**<0.001**		39.73	**<0.001**
11–20	17,464	49	52.0 (32.7–71.3)			NR		
20–40	14,221	69	81.0 (57.6–104.4)			NR		
≥40	6,347	142	23.0 (8.4–37.6)			28.0 (8.1–47.9)		
Unknown	1,367	65	6.0 (2.7–9.3)			7.0 (0.6–13.4)		
**Surgical procedure**								
No	1,074	113	6.0 (3.8–8.2)	83.66	**<0.001**		75.57	**<0.001**
Others	9,971	20	7.0 (0–20.1)			14.0 (3.8–24.2)		
Total thyroidectomy	52,558	211	66.0 (57.6–74.4)			85.0 (67.2–102.8)		
Unknown	480	3	NR			NR		
**Lymph node metastases**								
No	48,224	172	64.0 (54.4–73.6)	29.61	**<0.001**		34.41	**<0.001**
Yes	15,136	146	13.0 (6.7–19.3)			15.0 (7.7–22.3)		
Unknown	723	29	36.0 (28.0–44.0)			NR		
**Radiotherapy (radiation and/or isotopes)**								
No	33,350	83	12.0 (4.1–19.9)	21.67	**<0.001**		19.75	**<0.001**
Yes	29,556	254	52.0 (40.7–63.3)			58.0 (47.6–68.4)		
Refused	256	2	8.0 (NR)			8.0 (NR)		
Unknown	921	8	77.0 (NR)			77.0 (NR)		
**Histologic subtype**								
PTC	58,385	147	55.0 (41.8–68.2)	195.44	**<0.001**		203.07	**<0.001**
FTC	4,252	115	70.0 (49.7–70.3)			NR		
MTC	1,033	37	19.0 (11.1–26.9)			20.0 (11.5–28.5)		
ATC	413	48	2.0 (1.1–2.9)			2.0 (1.1–2.9)		
**Extraskeletal metastases**								
0 (no)	63,386	196	66.0 (51.3–80.7)	62.84	**<0.001**		68.00	**<0.001**
1	635	122	14.0 (8.6–19.4)			NR		
2 or 3	62	29	6.0 (4.3–7.8)			6.0 (4.3–7.8)		

PTC, papillary thyroid cancer; FTC, follicular thyroid cancer; MTC, medullary thyroid cancer; ATC, anaplastic thyroid cancer; OS, overall survival; TCSS, thyroid cancer-specific survival; NR, not reached.

**Figure 4:**
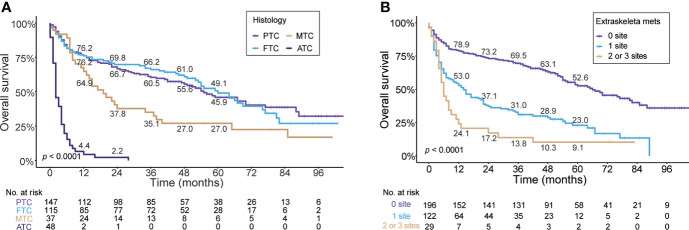
Kaplan–Meier’s overall survival curves are stratified by the histologic subtype **(A)** and the number of extraskeletal metastatic sites **(B)** in patients with bone metastases in newly diagnosed thyroid cancer. PTC, papillary thyroid cancer; FTC, follicular thyroid cancer; MTC, medullary thyroid cancer; ATC, anaplastic thyroid cancer.

In regard to histologic subtype, as depicted in **Figure 4A**, the Kaplan–Meier curve showed unfavorable prognosis in MTC (median OS, 19.0 months; 95% CI, 11.1–26.9) and ATC (median, 2.0 months; 95% CI, 1.1–2.9) patients with BM compared to PTC (median, 55.0 months; 95% CI, 41.8–68.2) and FTC (median, 70.0 months; 95% CI, 49.7–70.3) cases. The 5-year OS rates of MTC (27%) and ATC (0%) patients with BM were markedly less than those of PTC (45.9%) and FTC (49.1%) cases. Additionally, stratified by the number of extraskeletal metastatic sites (**Figure 4B** **)**, the patients without extraskeletal metastasis (namely those with BM alone) experienced the longest median OS (66.0 months, 95% CI, 51.3–80.7) and patients with extraskeletal metastasis to two or three sites experienced the shortest median OS (6.0 months, 95% CI, 4.3–7.8). The 5-year OS rate of patients with BM was 23.0% in patients with extraskeletal metastasis to one site and 9.1% in cases with two or three sites. In contrast, the 5-year OS rate was markedly higher (52.6%) in patients with BM alone.

**Figure 5:**
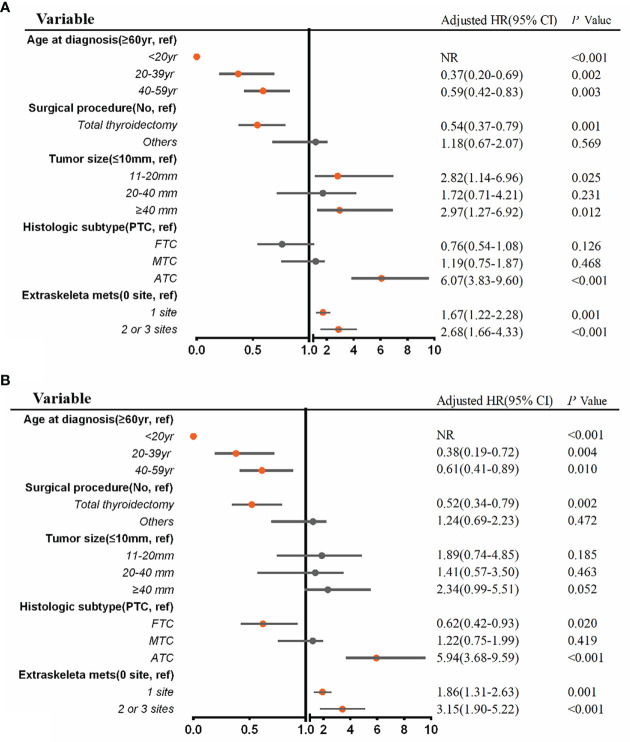
Multivariable Cox regression analysis for identifying independent risk factors of all-cause mortality **(A)** and thyroid cancer-specific mortality **(B)** among patients with bone metastases in newly diagnosed thyroid cancer. aHR, adjusted hazard ratio; CI, confidence interval; NR, not reached.

A multivariable Cox regression analysis for all-cause mortality among patients with BM at diagnosis of thyroid cancer was applied (**Figure 5A**). ATC (aHR, 6.07; 95% CI, 3.83–9.60; *p* <.001), extraskeletal metastasis to one site (aHR, 1.67; 95% CI, 1.22–2.28; *p* = .001) and extraskeletal metastasis to two or three sites (aHR, 2.68; 95% CI, 1.66–4.33; *p* <.001) were associated with an increased all-cause mortality. Meanwhile, age ≤ 20 years (aHR, NR; *p <*0.001), age 20 to 39 years (aHR, 0.37, 95% CI, 0.20–0.69; *p* = .002), age 40 to 59 years (aHR, 0.59; 95% CI, 0.42–0.83; *p* = .003), and total thyroidectomy (aHR, 0.54; 95% CI, 0.37–0.79; *p* = .001) were significantly associated with a decreased all-cause mortality. Additionally, multivariable Cox regression analysis for thyroid cancer-specific mortality among patients with BM in newly diagnosed thyroid cancer is also shown in **Figure 5B**.

## Discussion

Thyroid cancer is predisposed to BM, ranking third in the incidence of BM following breast and prostate cancer (1). However, the incidence of BM varies widely in the literature, likely attributable to biases among the reports and the existent data being limited to older, retrospective, single-institution studies with small cohorts mainly focused on differentiated thyroid cancer (3–5). Additionally, BM occurs more frequently in FTC and MTC patients than in PTC patients, and cases with BM are at greater risk of overall mortality and cancer-specific mortality (10, 16, 17), stressing the importance of early detection of BM at diagnosis, especially diligently in more aggressive subtypes of thyroid cancer.

In this SEER study, distant metastasis was found in 1.7% of thyroid cancer patients at the time of diagnosis, comparable to 2.03% (for PTC) as reported by Toraih et al. (18) and 1.045% (for DTC) by Qi et al. (6) However, an autopsy study conducted from the nationwide Duch pathology registry reported distant metastasis was present in up to 35.1% of thyroid cancer patients, and the majority of patients had more than one metastasis (19). BM occurred in 0.5% (347/64083) of all newly diagnosed thyroid cancer patients, which was lower than previous studies as reported ~4% (20). Among the subset of patients with metastases, BM most likely occurred in those with FTC histologic type, with the incidence proportion up to 61.5%. The strengths of this study are the population-based setting, the inclusion of four histologic subtypes of thyroid cancer, and the large sample size; hence, our results are generalized to the population. Nevertheless, the shortcoming, as in our study, was the unavailability of information regarding the incidence of BM that developed at any time over the disease course as opposed to at first presentation, and patients in the entire cohort were likely diagnosed as a result of bone pain given that screening imaging during perioperative management or follow-up for the possible BM is not routinely considered due to lack of proven benefit. Hence, the true incidence of BM in thyroid cancer patients is likely underestimated by the results of this study.

Exploring risk factors or molecular biomarkers of distant metastases for thyroid cancer is critical for the sake of early diagnosis and interventions. Previous studies focused on the clinicopathological characteristics reported that older age (14, 15, 21), male gender (21, 22) higher grade or T stage (14, 15), black race (15), large tumor size (21, 22), and lymph node metastases (22, 23) were independent risk factors to have BM. Recently, a few studies have revealed that gene mutations (RAS, BRAF, or TERT), especially TERT promoter mutations (24, 25) and gene fusions (RET, ALK, or NTRK1) (26), HOXD9 oncogene (27), and chromosome 22q loss (26) could serve as molecular biomarkers for predicting distant metastases for DTC. Moreover, coexisting BRAF V600E and TERT C228T mutations form a novel genetic background for the most aggressive subgroup of PTC and had the worst clinicopathologic outcomes (28). Among the entire cohort in our study, patients of the male gender, black race, older age, larger tumor size, and lymph node metastases had significantly greater odds of having BM at initial diagnosis after controlling for sociodemographic factors and clinical and pathologic characteristics. However, we did not find black or male patients with BM had worse median OS compared to white race or female patients. In advance, similar to the results described before (9, 10, 29), wherein they reported patients with FTC and MTC were more likely to develop BM, our results revealed that FTC (aOR, 6.33), MTC (aOR, 6.04), and ATC (aOR, 6.21) subtypes were associated with significantly greater odds of having BM at diagnosis. Therefore, closer bone surveillance in patients with these histologies is required.

Prior studies done largely on DTC reported that older age (30), tumor size (21), male gender (31), radioactive iodine resistance (11, 32), metastasis, and higher tumor grade (18, 33) were associated with greater mortality, while lymph node involvement (18) at presentation did not show a significant influence on mortality. Similarly, in our study, we found that larger tumor sizes or older age were associated with poorer OS. Moreover, we also found that radiotherapy procedure and lymph node involvement status at initial diagnosis did not demonstrate a significant effect on OS after adjustment.

Since SEER data include approximately 30% of the United States population (34), the incidence and median survival we describe are highly generalizable and likely more reflective of the population experience compared with previously published data focused primarily on patients treated at a single institution (5). In this study, the median survival of patients with BM at presentation was 40.0 months, which, however, varied significantly by histologic subtype, ranging from 2.0 months in ATC patients to 70.0 months in FTC patients. The 5-year OS rates were 45.9% and 49.1% for PTC and FTC patients with BM at diagnosis compared to 27% for MTC cases. Therefore, interestingly, although the incidences of BM in FTC and MTC patients (2.7% and 3.6%, respectively) were significantly higher than that in PTC (0.3%) at initial diagnosis, there were no significant differences for OS between the patients with BM using multivariable Cox regression analysis (FTC vs. PTC, *p* = 0.126; MTC vs. PTC, *p* = 0.468), indicating that the tumor subtype (PTC, MTC, or FTC) did not seem to significantly affect the prognosis of patients with BM, which was similar to the previous report (16). A possible explanation of this finding is that in comparison with FTC patients, who are more likely to have distant metastases by the bloodstream, PTC cases are often at an advanced stage when BM is detected at the moment of diagnosis, while the prognosis of MTC is poorer regardless of the occurrence of BM. Of course, the shorter observation period of this study may also be an important factor.

Of the 347 thyroid cancer patients with BM at presentation in our SEER study, 196 patients (56.5%) had BM alone, 122 patients (35.2%) with one extraskeletal metastasis to a distant site (lung, brain, or liver), and 29 patients (8.3%) with two or three extraskeletal metastatic sites. We found that thyroid cancer patients with BM alone at diagnosis were associated with the longest survival (median survival, 66.0 months), while patients with two or three extraskeletal metastatic sites experienced the poorest survival (median survival, 6.0 months). Contrary to the findings by Toraih et al. (18), wherein they reported a 5-year survival rate of 25% for patients with BM in PTC, our results demonstrated the 5-year OS rate from diagnosis for thyroid cancer patients with BM was 38.7%, consistent with 38% reported by Califano et al. (35). Moreover, the 5-year OS rate was 52.6% in patients without extraskeletal metastases, which, however, drastically declined to 23.0% in patients with one extraskeletal metastasis and 9.1% with two or three extraskeletal metastatic sites. Consistent with our results, Sampson et al. (36) reported the 3-year survival of single bone metastasis to be 56%. Wang et al. (12) reported that the 5-year survival rate in patients with metastasis limited to one organ was 77.6%, while that in patients who develop second organ involvement was as low as 15.3%. These survival variations could be probably due to the differences in the study population, as well as treatment strategies and accessibility for tumor eradication. In advance, after adjustment, patients with one extraskeletal metastasis (aHR, 1.67) and cases with two or three extraskeletal metastatic sites (aHR, 2.68) were associated with a significantly less favorable prognosis compared to those without extraskeletal metastases. Our work suggests it will be important to identify patients in whom extraskeletal metastases may evolve because extraskeletal metastases are common in patients with BM at diagnosis of thyroid cancer but have an obvious poorer prognosis.

Our study, however, had inherent limitations in the context. First, we only had information about the presence or absence of BM at the initial diagnosis of thyroid cancer. The SEER database does not provide information about disease recurrence or subsequent sites of disease involvement. Nevertheless, some patients might subsequently develop BM later in the disease course, which cannot be captured and analyzed in our study. Second, since screening imaging is not routinely recommended during perioperative management or follow-up for the possible BM due to lack of proven benefit, patients with BM are likely to have symptomatic bone pain at the initial diagnosis of thyroid cancer. Hence, the actual incidence proportion of BM is in fact underestimated. Third, the patient’s related information about molecular biomarkers such as gene mutations and fusion, profession, performance status and comorbidities, and treatment patterns such as targeted therapy, which have an impact on survival and clinical outcomes, is not recorded in the SEER database.

## Conclusion

Taken together, our large population-based study using the SEER database provides generalized insight into the epidemiology of BM in patients with newly diagnosed thyroid cancer. Patients with FTC, MTC, and ATC at diagnosis have a greater likelihood of developing BM, lending support to proactive surveillance of patients with these histologies and early detection, intervention, and making treatment strategies for patients with BM to improve their survival rates. Additionally, we should be aware of and give special attention to patients with extraskeletal metastases at the initial diagnosis of thyroid cancer due to their obvious poorer survival time.

## Data availability statement

The raw data supporting the conclusions of this article will be made available by the authors, without undue reservation.

## Author contributions

Conceptualization: RZ and WXZ. Methodology: QJ and CW. Software: WXZ and JC. Formal analysis: WXZ. Resources: RZ and JT. Data curation: RZ and WXZ. Writing—original draft: RZ. Supervision: WZ and JT. Project administration: JT and ZM. The final draft was read and approved by all authors. All authors listed have made a substantial, direct, and intellectual contribution to the work and approved it for publication.

## Funding

The work was supported by the National Natural Science Foundation of China (grant: 81801732) and the Project from New Century Excellent Talent of Tianjin Medical University General Hospital (awarded to RZ).

## Acknowledgments

We thank Mr. Jing Yuan (Department of Biostatistics, CSPC Zhongqi Pharmaceutical Technology (Shijiazhuang) Co., Ltd., Hebei, China) for the appropriate statistical analysis of the data and the helpful interpretation of the results.

## Conflict of interest

The authors declare that the research was conducted in the absence of any commercial or financial relationships that could be construed as a potential conflict of interest.

## Publisher’s note

All claims expressed in this article are solely those of the authors and do not necessarily represent those of their affiliated organizations, or those of the publisher, the editors and the reviewers. Any product that may be evaluated in this article, or claim that may be made by its manufacturer, is not guaranteed or endorsed by the publisher.
